# Synthesis of Metal-Loaded Carboxylated Biopolymers with Antibacterial Activity through Metal Subnanoparticle Incorporation

**DOI:** 10.3390/antibiotics11040439

**Published:** 2022-03-24

**Authors:** Farzaneh Noori, Meriem Megoura, Marc-André Labelle, Mircea Alexandru Mateescu, Abdelkrim Azzouz

**Affiliations:** 1 Nanoqam, Department of Chemistry, Faculty of Sciences, Université du Québec à Montréal, Montreal, QC H3C 3P8, Canada; noori.farzaneh@courrier.uqam.ca (F.N.); megoura.meriem@courrier.uqam.ca (M.M.); labelle.marc-andre.2@courrier.uqam.ca (M.-A.L.); 2 École de Technologie Supérieure, Montreal, QC H3C 1K3, Canada

**Keywords:** carboxymethyl starch, carboxymethyl cellulose, metal subnanoparticles, antibacterial activity

## Abstract

Carboxymethyl starch (CMS) and carboxymethyl cellulose (CMC) loaded by highly dispersed metal subnanoparticles (MSNPs) showed antibacterial activity against *E. coli* and *B. subtilis* strains. Copper and silver were found to act in both cationic and zero-valence forms. The antibacterial activity depends on the metal species content but only up to a certain level. Silver cation (Ag^+^) showed higher antibacterial activity as compared to Ag^0^, which was, however, more effective than Cu^0^, due to weaker retention. The number of carboxyl groups of the biopolymers was found to govern the material dispersion in aqueous media, the metal retention strength and dispersion in the host-matrices. Cation and metal retention in both biopolymers was found to involve interactions with the oxygen atoms of both hydroxyl and carboxyl groups. There exists a ternary interdependence between the Zeta potential (ZP), pH induced by the biocidal agent and its particle size (PS). This interdependence is a key factor in the exchange processes with the surrounding species, including bacteria. Clay mineral incorporation was found to mitigate material dispersion, due to detrimental competitive clay:polymer interaction. This knowledge advancement opens promising prospects for manufacturing metal-loaded materials for biomedical applications.

## 1. Introduction

Excessive use of conventional organic antibiotics was already recognized as favoring pathogenic microorganism resistance with negative impact on animal and human health [1,2,3,4]. Nowadays, this is regarded as being a major environmental issue. The use of highly dispersed metals could be an interesting avenue to explore, since metal nanoparticles (MNPs) have a broad-spectrum activity being efficient against both Gram-positive and Gram-negative microorganisms [5,6]. Their non-selective toxicity arises from the production of a wide variety of reactive oxidative species (ROS) through the oxidative stress induced on microorganisms.

Among many other metals, copper and silver already showed antibacterial activity [6,7,8,9,10,11] and anticancer properties [12]. These features were found to be improved by high contact surface with microorganisms, but their tendency to aggregate [13,14,15] is known to affect their toxicity toward bacteria [16,17]. High dispersion into the smallest particles possible by using suitable host-matrices or surfactants bearing highly chelating groups can prevent this drawback [6,18,19,20,21,22,23,24,25,26]. Therefore, the synthesis of metal subnanoparticles by using carboxymethylated biopolymers was the target of the present work. The novelty core resides less in the synthesis of metal subnanoparticles than in an original approach to correlate the interactions involved between the incorporated metal and the host-matrices with their antibacterial activity. Such an approach has not been tackled so far.

Biocompatibility is an essential requirement for such dispersing agents, and carboxymethyl starch (CMS) and carboxymethyl cellulose (CMC) are low-cost and interesting polysaccharides for such a purpose. They are commonly used as drug carriers and other biomedical applications, owing to their modification capability, non-toxicity, high biodegradability and biocompatibility and pH sensitivity [27,28,29,30,31,32,33,34]. Upon chemical functionalization, they acquire a different number of carboxymethyl (CM) groups that can capture metal cation via ion exchange. The CM oxygen atoms should also provide electron pairs that can stabilize MNPs, preventing their aggregation via Lewis acid–base interactions [35]. Similar interactions were already reported for hydroxyl groups of polyol dendrimer and clay minerals [6,36,37,38,39].

However, in contrast to hydroxyls, which display amphoteric to weak basic character, CM groups are rather slightly acidic, displaying a pH-dependent behavior. The latter is, in turn, expected to influence the metal retention strength and the dispersion of the metal within the host-matrices and that of the biopolymer in aqueous media. These key factors should influence not only the contact surface of the metal-loaded biopolymer with both the aqueous media and dispersed microorganisms but also a possible metal release that seems to govern the antibacterial activity [6]. The study of these influences is the main objective of the present work.

Increasing the degree of substitution (DS) defined as the number of carboxyls per glucose unit is expected to favor metal dispersion through particle size reduction. In the meantime, a high number of carboxyl groups is assumed to enhance the effect of pH on the aggregation–dispersion of the biopolymer entanglement in bacteria-infected media. Metal–cell interaction is known to result in an oxidative stress that unavoidably produces acidic species and fluctuations in metal-loaded biopolymer compaction-porosity grade that control the contact surface. 

This interaction must also be influenced by competitive ones arising from any HO-bearing species, including water molecules and cell membrane. That is why a special interest was devoted to the role of solid particles bearing surface hydroxyl groups. For this purpose, CMS, CMC and their combinations with sodium montmorillonite (NaMt) were loaded with copper or silver particles. Comprehensive characterization through measurements of the zeta potential and particle size, as well as analysis via X-Ray Photoelectron spectroscopy (XPS), Transmission Electron Microscopy (TEM) and Energy-Dispersion X-Ray Fluorescence (ED-XRF), appears to be a judicious approach in this regard. The antibacterial activities of the host-matrices before and after combination with the clay mineral and incorporation of Cu^0^ and Ag^0^ nanoparticles were evaluated in terms of inhibition zone diameter (IZD) on Gram-positive (*B. subtilis S*168) and Gram-negative (*E. coli DH5α*) bacterial species and correlated to the material features. Moreover, viability tests were carried out on *E. coli* as a representative model.

## 2. Materials and Methods 

### 2.1. Chemicals and Materials

Copper acetate, Cu(CH_3_COO)_2_, silver nitrate, AgNO_3_ and sodium borohydride, NaBH_4_ 98% were supplied by Fisher chemicals, Canada. Sodium carboxymethyl cellulose (CMC) with a degree of substitution (DS) of 0.92 ± 0.01 carboxymethyl group (CM) per glucose unit (Gu) and 90 kDa MW was purchased from Sigma Aldrich, Canada. Sodium carboxymethyl starch (CMS) with a DS value of 0.51 ± 0.01 CM/Gu and 100 kDa MW was prepared by treatment of High Amylose Starch (Hylon VII) with sodium monochloroacetate. The degree of substitution of the hydroxyls by the carboxymethyl groups was measured by back titration, as previously described [40]. For this purpose, an aliquot of CMC or CMS was fully protonated in 2 M aqueous HCl solution for 30 min, then filtered and washed with distilled water. Total disappearance of the FTIR 1590 cm^−1^ IR band provided evidence of a total conversion of carboxylate into carboxylic acid. The DS of each carboxymethylated biopolymer was determined by titrating 100 mg of biopolymer previously dissolved in 0.05 M aqueous NaOH solution with 20 mL of 0.05 M aqueous HCl solution, using phenolphthalein as indicator [41,42]. The investigated biopolymers (Supplementary Figure S1) were used without further purification. Lysogeny Broth medium and agar were supplied by Biobasic (Markham, ON, Canada). *Escherichia coli DH5Alpha* and *Bacillus subtilis S168* bacterial strains were purchased from American Type Culture Collection (ATCC, Manassas, VA, USA). Na^+^-montmorillonite (NaMt) used in combination with the investigated biopolymers was prepared through purification of an Aldrich bentonite according to the same procedure as previously reported [6].

### 2.2. Preparation of Cation- and Metal-Loaded Materials

Cation-loaded CMC samples (Cu^2+^/CMC and Ag^+^/CMC) with 1 mmol/g cation content were prepared by adding dropwise 10 mL of 0.1 mol/L Cu(CH_3_COO)_2_ or AgNO_3_ solutions in CMC solutions (1 g CMC in 50 mL water) at 50–60 °C under stirring for 3 h. Further addition of 4 mL of 0.5 mol/L NaBH_4_ under nitrogen stream for 10 min resulted in MNP-loaded CMC (Cu^0^/CMC or Ag^0^/CMC) with 1 mmol/g metal content through cation reduction into zero-valent metal (ZVM). Given that the DS value of CMC is 1.8 times higher than that of CMS, cation-loaded CMS samples (Cu^2+^/CMS and Ag^+^/CMS), the addition of 250 mg of CMS to 10 mL of 0.01 mol/L Cu(CH_3_COO)_2_ or 0.01 mol/L AgNO_3_ aqueous solutions under vigorous stirring at 25 °C for 1 h resulted in lower cation content of 0.4 mmol/g. Both mixtures were sonicated for 50 min (500 W, 20 kHz) at room temperature for homogenization. Consecutive cation reduction by adding 10 mL of 0.02 mol/L NaBH_4_ resulted in MNP-loaded CMS (Cu^0^/CMS and Ag^0^/CMS). The metal polarizing effect was investigated through zeta potential measurements of various CMC and CMS samples previously treated with aqueous solutions of other cations, such as Zn^2+^, Co^2+^, Pt^2+^, Ti^2+^ and Au^+^. Biopolymer/clay composites (CMC/NaMt and CMS/NaMt) with 1:1 Wt. ratio were prepared by previous dispersion of 0.5 g of CMC or CMS in 50 mL distilled water at 50–60 °C and slow addition of an aqueous suspension containing 0.5 g of NaMt under vigorous stirring for 1 h. Cu^2+^/CMC/NaMt, Ag^+^/CMC/NaMt, Cu^2+^/CMS/NaMt and Ag^+^/CMS/NaMt were obtained by adding 10 mL of 0.1 mol/L Cu(CH_3_COO)_2_ or AgNO_3_ at room temperature under vigorous stirring for 3 h. Consecutive reduction of Cu^2+^ or Ag^+^ by adding 4 mL of 0.5 mol/L NaBH_4_ gave rise to Cu^0^/CMC/NaMt, Ag^0^/CMC/NaMt, Cu^0^/CMS/NaMt and Ag^0^/CMS/NaMt. All samples were dried by lyophilization and stored in a sealed desiccator containing O_2_-free dry nitrogen.

### 2.3. Material Characterization

The effects of the metal dispersion in both biopolymers and their NaMt-composites on the behavior of their suspensions in aqueous media were assessed in terms of surface charge and average particle size in correlation with the induced pH. This was achieved through triplicate measurements of the Zeta potential (BrookHaven Instrument Corp., ZetaPlus/Bl-PALS, Holtsville, NY, USA) and *Dynamic Light Scattering* measurements (DLS) (Malvern, Zetasizer Nano S90, Worcestershire, UK), respectively. Deeper insights in MNP incorporation were achieved through Transmission Electron Microscopy (TEM) and Energy-Dispersion X-Ray Fluorescence (ED-XRF), using a JEOL JEM-2100F equipment (with an accelerating voltage of 200 kV) coupled to an EDAX X-ray fluorimeter. The samples were previously dispersed in methanol and dried on holey carbon-coated Ni grids. The ED-XRF spots were denoted as ***eds1*** and ***eds2***.

Additional analyses were performed by X-Ray Photoelectron Spectroscopy (Thermo Scientific K-Alpha) XPS of 400 µm spots and a monochromatic Aluminum-kα X-ray source (260 watts in constant pass energy mode in two 200 eV and 1 eV steps). High-resolution scans (Ag_3d_, C_1s_, O_1s_ and Cu_2p_) were recorded at a constant pass energy of 50 eV and 0.1 eV steps. XPS data were processed by using the software Thermo Avantage. Deconvolution was applied for all symmetric and asymmetric XPS signals for detecting potentially overlapped peaks for the key-element interacting with cations and zero-valent metals (Supplementary Figures S2–S5). 

### 2.4. Antibacterial Tests

*Bacillus subtilis S168* and *Escherichia coli DH5α* were cultivated in Lysogeny Broth (LB) overnight in an incubator shaker at 37 °C, 100 rpm. The antimicrobial behavior of the as-prepared samples was first evaluated in terms of inhibition zone diameter (IZD) for two bacteria strains in 10 cm–diameter Petri dishes. The latter were pre-inoculated with approximatively 74 × 10^6^ CFU.mL^−1^ of each strain with an optical density at 600 nm (OD_600_) of 0.5 as measured by a UV–Vis spectrophotometer (Biochrom Libra S50 UV/VIS Instrument). A preliminary test was performed by seeding 1 g of each lyophilized sample directly into the center of the as-prepared Petri dishes. All experimental runs were performed in triplicate at 37 °C for 24 h. The influence of various variables (pH and metal contents) was evaluated through impregnation of the discs of blotting paper with aqueous suspensions of similar amounts of metal cation-loaded biopolymer but with different metal contents (0.1, 0.2, 0.5, 1.0, 2.0 and 5.0 mmol/g biopolymer). Another set of samples was prepared by mixing the same amount of metal-loaded biopolymer with similar metal content of 1 mmol/g biopolymer) at different pH (1, 3, 5, 7 and 9) adjusted with HCl or NaOH aqueous solutions. The discs were placed on the pre-inoculated agar and incubated for 24 h at 37 °C. Negative controls were performed in each assay. As the inhibition zones (IZ) were not circular, the area of the inhibition zone was estimated by using Image J software for a 10 cm scale (Supplementary Figure S6). The average IZD and standard deviation were assessed through triplicate graphical measurements. Additional evaluation of the antibacterial capacity was performed by using silver and copper-loaded CMC materials with *E. coli* as bacterial model in LB broth with an OD_600nm_ of 0.5. The nanoparticles were added to the *E. coli* suspension at two different concentrations (0.1 and 1 mg/mL) and incubated at 37 °C, 180 rpm for 24 h. During this period, sampling was pre-elevated at 3, 6, 9 and 24 h. Then dilution series were carried out, and 0.1 mL was spread on LBA Petri dishes. The plates were then incubated at 37 °C for 24 h, and CFUs were counted.

## 3. Results and Discussion 

### 3.1. Biopolymer and Composite Behavior in Aqueous Media 

#### 3.1.1. Effects of Biopolymer Structure and Clay Addition 

The starting CMC displayed a higher zeta potential (ZP) value (−33.83 mV) as compared to CMS (−26.11 mV), due to a higher number of weakly acidic CM groups expressed in terms of the degree of substitution (DS) and also reflected by a slightly lower pH (6.37 versus 6.50) (Table 1). As expected, this resulted in a higher dispersion of CMC due to stronger electrostatic repulsion forces, as suggested by the lower particle size (289.3 nm for CMC versus 350.2 nm for CMS). Montmorillonite incorporation was found to raise the ZP up to −48.36 for CMC/NaMt and up to −33.42 for CMS/NaMt, and, subsequently to reduce the particle size down to 266.6 nm and 299 nm, respectively. This can be explained by additional contributions of the exchangeable sites and deprotonated silanol groups of the clay mineral. This is in agreement with the slight pH decrease from 6.37 and 6.56 down to 6.32 and 6.41, respectively.

#### 3.1.2. Effect of Metal Incorporation

The incorporation of copper and silver cations into CMC and CMS induced a marked ZP decrease from −33.83 mV (CMC) and −26.11 mV (CMS) down to −13.82 mV for Cu^2+^/CMC and −13.28 mV for Cu^2+^/CMS, as compared to −30.39 mV for Ag^+^/CMC and −17.9 for Ag^+^/CMS. The simultaneous pH decrease, more pronounced with Cu^2+^ ions, must be due to the Bronsted acidity induced by both cations retained by ion exchange and/or Lewis acid–base interactions (LAB) with the electron pairs of the oxygen atoms of the CM groups. The stronger effect of the Cu^2+^ cation can be explained by its higher capacity to dissociate the surrounding water molecules as compared to Ag^+^ (Reaction (1)). The consecutive H^+^ release unavoidably leads to carboxyl protonation and ZP decrease (Reaction (2)).

(1) $${\left[M,\;{\mathrm{nH}}_{2}O\right]}^{2+}⇋{\left[M,\;\left(n-1\right)H_{2}O,\;\mathrm{OH}\right]}^{+}+H^{+}⇌\left[M,\;\left(n-2\right)H_{2}O,\;\mathrm{OH}\right]{}^{\circ}+H^{+}\;$$

(2) $$\mathrm{Biopolymer}-\mathrm{COO}{\mathrm{COO}}^{-}\;\to \mathrm{Biopolymer}-\mathrm{COOH}\;\mathrm{Protonation}\;$$

**Figure 1:**
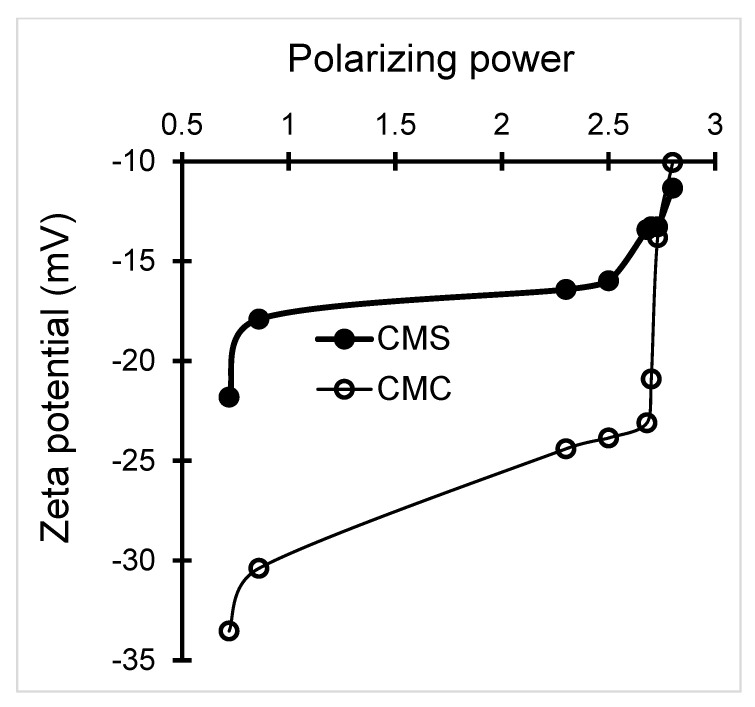
Influence of the polarizing power of the incorporated metal cation on the Zeta potential of metal-loaded CMC and CMS. The Zeta potential was measured for aqueous suspensions of CMC and CMS treated with aqueous solutions of different metal cations (Cu^2+^, Ag^+^, Zn^2+^, Co^2+^, Pt^2+^, Ti^2+^ or Au^+^).

This is in agreement with the higher polarizing power (PP) of Cu^2+^ (Supplementary Table S1). This factor was found to reduce the ZP for both metal-loaded biopolymers regardless of the incorporated cation (Figure 1). Here, the polarizing power is the cation capacity to attract the electron cloud of pseudo-anioninc OH groups in surrounding water molecules. Their dissociation leads to an enhancement OH-anions retention by the hydrated cation and to proton release that reduces the pH. 

Less accentuated pH decreases down to 5.66–6.00 and 5.9–6.00 were observed after cation incorporation into CMC/NaMt and CMS/NaMt samples, most likely due to a compensating effect of CM group protonation at this pH value close to the pKa value. In contrast cations, Zero-Valent Metals (ZVMs) rather induced ZP increases from −33.83 to −44.05 for Cu^0^/loaded CMC and −54.53 for Ag^0^/CMC, and from −26.11 up to −31.36 for Cu^0^/loaded CMS and −40.16 for Ag^0^/CMS. Here, MNP interaction with the electron pairs of the oxygen atoms in CM groups are expected to mitigate proton release, as reflected by weaker pH decrease down to 6.0 (Cu^0^/CMC/NaMt), 6.0 (Ag^0^/CMC/NaMt), 6.37 (Cu^0^/CMS/NaMt) and 6.0 (Ag^0^/CMS/NaMt).

#### 3.1.3. Zeta Potential–pH–Material Particle Size Interdependence

**Figure 2:**
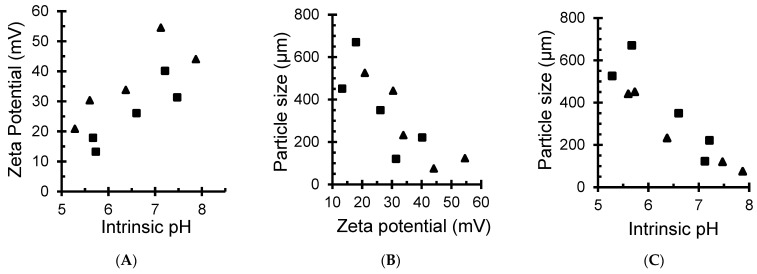
Relationship between Zeta potential and pH (**A**), material particle size and Zeta potential (**B**) and material particle size and pH (**C**) of the dispersion media. In black square: CMC-based samples, including CMC, Cu^2+/^CMC, Cu^0^/CMC, Ag^+^/CMC and Ag^0^/CMC. In black triangle: CMS-based samples, including CMS, Cu^2+/^CMS, Cu^0^/CMS, Ag^+^/CMS and Ag^0^/CMS.

Changes in ZP and pH are almost linearly interdependent (Figure 2A). In the meantime, increasing ZP enhances the repulsion forces and material dispersion that results in a decrease in material particle size (Figure 2B). Subsequently, high intrinsic pH of the aqueous media appears to favor material dispersion through a decrease in particle size, as shown in Figure 2C.

This ternary interdependence is of great importance because it clearly demonstrates that the surface basicity of the antibacterial agent is a key-factor in the improvement of the contact surface and exchange processes with the targeted microorganisms. CMC and CMS and their NaMt-based composites have pH-sensitive chemical functions. The acid-base character can be modulated by the surface density of the CM groups, lattice oxygen atoms, in-plane and out-of-plane silanols, using suitable exchangeable cations.

### 3.2. XPS Assessments of Induced Interactions

#### 3.2.1. XPS Evidence of Cation-Matrix Interactions

XPS measurements revealed noticeable changes in O_1s_ XPS signal after the incorporation of metal cation and MNP in both carboxymethylated biopolymers. The changes were reflected by higher signal intensity of 40,000–100,000 counts per second (CPS) for metal-loaded CMC (Supplementary Figures S2 and S3) as compared to 15,000–75,000 CPS for metal-loaded CMS (Supplementary Figures S4 and S5). This can be explained by the higher DS of 0.92 ± 0.01 for CMC versus 0.51 ± 0.01 for CMS, providing evidence of the involvement of the oxygen atoms of the CM groups in metal capture and stabilization. Weaker O_1s_ signal intensity was observed after Cu^2+^ incorporation as compared to Ag^+^, suggesting stronger compaction of the polymer entanglement that hinders electron release. This can be explained by the sandwiching effect of the positive charges of bivalent cations shared between two next-neighboring CM groups. Deeper insights into the O_1S_ XPS signals revealed BE shift for H-O-C:Cu^2+^ interaction of 1.3 eV in CMC (Supplementary Table S2) and 1.0 eV in CMS (Supplementary Table S3), i.e., stronger Cu^2+^ retention by H-O-C groups of CMC carboxyls (Table 2). These values are at least three times higher than those registered with Ag^+^ cation (0.3 in both CMC and CMS), indicating a much stronger retention of Cu^2+^ in both biopolymers as compared to silver.

As a general tendency, C_1s_ signals of C-O-C of glucose ring, C-C and O-C=O groups showed barely detectable BE shift not exceeding −0.3 eV in CMC (Supplementary Table S2) and −0.5 eV in CMS (Supplementary Table S2). This result is of great importance, because it demonstrates that (i) the carbon atoms do not directly interact with the incorporated metal species; and (ii) weak BE shifts, if any, are induced by next-neighboring oxygen atoms of the OH and carboxyl groups of both biopolymers.

Here, also, the intensity decreases in C_1s_ XPS signals in CMC must be due to structure compaction that reduces electron release, leading to signal mitigation [36,39]. Surprisingly, CMS showed more pronounced C_1s_ intensity decay in spite of its lower density of CM groups, suggesting accentuated biopolymer winding around the metal cation or MNP encapsulated. Cation retention and stabilization by carbonyl groups (C=O), if any, should involve only weaker interactions. This is supported by the lower BE shift observed for the O_1S_ signals of Cu^2+^:O=C interaction (0.4 eV in CMC and 0.7 eV in CMS) and Ag^+^:O=C interaction (0.5 eV in both CMC and CMS).

#### 3.2.2. Biopolymer Interactions with MNP and Montmorillonite

MNP retention by the biopolymers appears to involve stronger -O:metal interaction as compared to metal cation, given that the CuNP insertion induced higher O_1s_ BE shift of 0.79 eV and 1.69 eV for Cu^0^:O=C and Cu^0^:H-O-C interactions in CMC and of 1.3 eV and 1.50 eV for Ag^0^:O=C and Ag^0^:H-O-C interactions in CMS (Supplementary Table S3). These values also indicate the stronger interaction with Cu^0^ and Ag^0^ by hydroxyl groups than by C=O in the carboxyl groups. A similar phenomenon was noticed for Cu^0^, but the reverse sequence was observed for Ag^0^ in CMS, presumably due to a higher hydroxyl affinity toward CuNP, as compared to C=O in both polymers. Interestingly, AgNP appears to selectively interact more with hydroxyls in CMC, but with C=O in CMS, thereby confirming the key role of the biopolymer structure. 

NaMt/CMC and NaMt/CMS showed no noticeable BE shift of Si2s, Si2p and Al2p XPS signals after Cu^2+^ and Ag^+^ incorporation, as already reported elsewhere [6]. This is presumably because cation retention involves mainly ion exchange. This is even more plausible for the incorporated cation amount (1 mmol/g in CMC samples and 0.4 mmol/g in CMS), which is, by far, lower than the total cation-exchange capacity of the NaMt-modified biopolymers. However, the addition of NaMt to CMC and CMS induced similar XPS signals to those reported for MNP interaction with silanols and Si-O-Si groups [6]. This was illustrated by the BE shift of the O_1s_ signal from 532.6 (NaMt) down to 531.5 eV (Cu^0^) and 531.6 (Ag^0^). A more pronounced BE shift was registered for the Si2s XPS signal from 154.55 eV to 152 eV (Cu^0^) and 149 eV (Ag^0^). No BE shift was noticed for Al2p XPS, suggesting, at most, a negligible MNP:Al-OH interaction.

### 3.3. Zero-Valent Metal Dispersion

**Figure 3:**
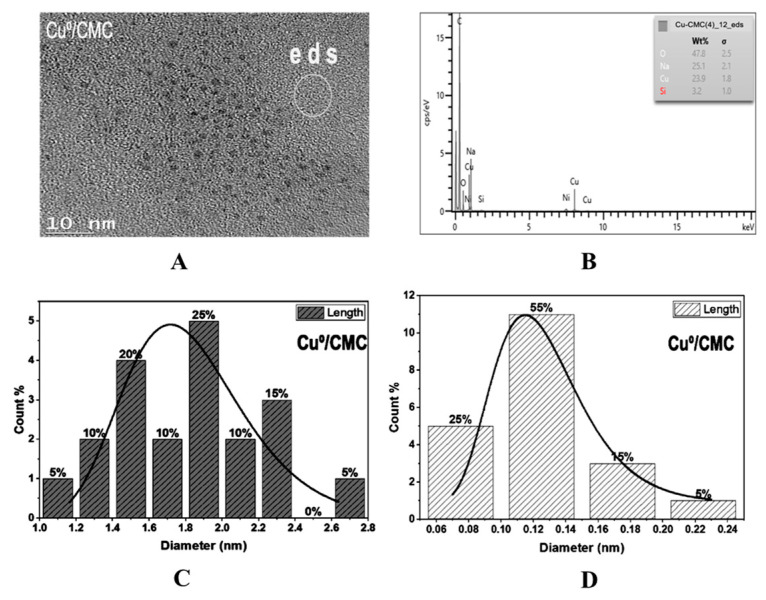
TEM image (**A**), ED-XRF spectrum (**B**) and Cu^0^ particle size distribution in CMC (**C**,**D**).

**Figure 4:**
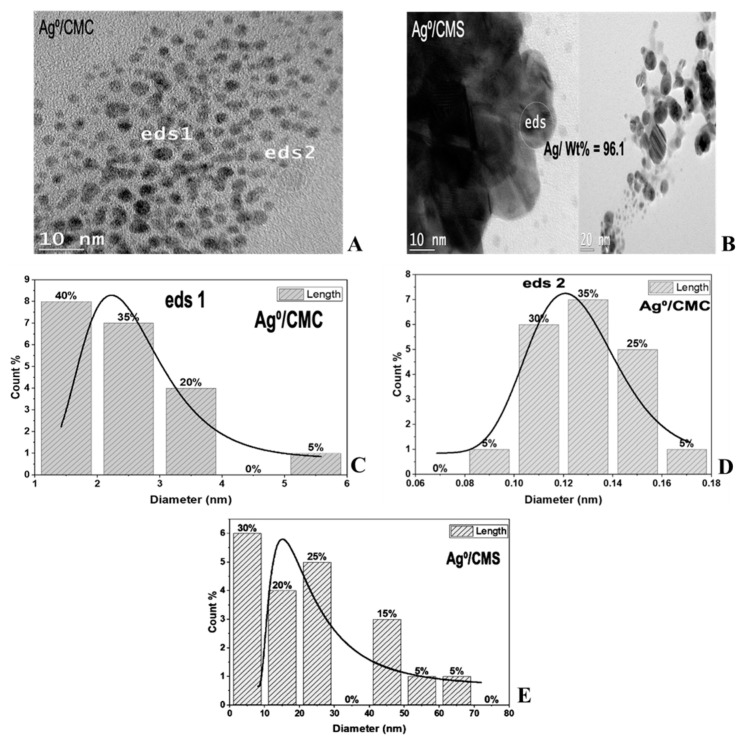
TEM images (**A**,**B**) and Ag^0^ particle size distribution in CMC (**C**,**D**) and CMS (**E**).

The TEM image of Cu^0^/CMC displayed a uniform dispersion of pseudo-spherical 1–3 nm CuNP (Figure 3A,C) and of subnanometric particles not exceeding 0.3 nm (Figure 3B,D). Ag^0^/CMC showed a particle size below 6 nm, mostly (ca. 95%) below 4.0 nm, including 40% of 1.0–2.0 nm particles (Figure 4A,B). Such a high dispersion must be due to the high density of potential chelating sites in CMC. Deeper insights through Energy-Dispersive X-Say Spectroscopy (ED-XRF) of two different spots located in an apparently void area between AgNP (Figure 4A,C) revealed AgNP contents of 84.3 wt.% in spot 1 and 28 wt.% in spot 2. This provides clear evidence of the occurrence of Ag^0^ subnanoparticles (AgNP) barely detectable by TEM. Particle size assessment using Image-J software on magnified spot eds2 confirmed the existence of subnanometric AgNP in the range of 0.08–0.1 nm (Figure 4D). Here, the higher CM group density of CMC appears to be responsible for its higher dispersion capacity as compared to CMS. As expected, bulkier MNPs were obtained in CMS, since ca. 25% of the incorporated AgNP displayed a particle size ranging from 40 to 70 nm (Figure 4E).

**Figure 5:**
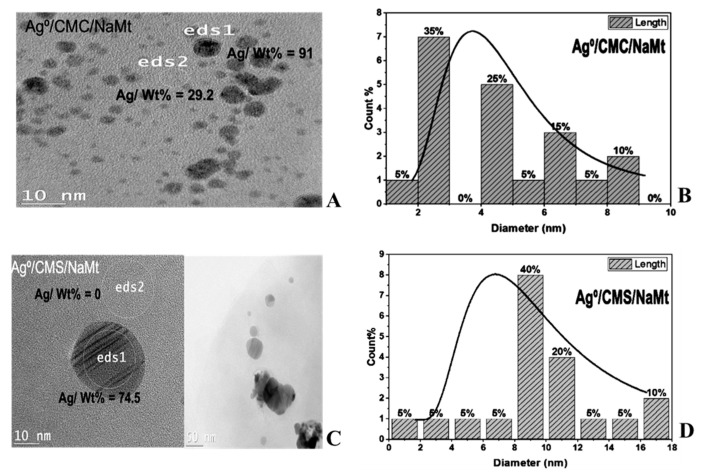
TEM images and Ag^0^ particle size distribution in CMC/NaMt (**A**,**B**) and CMS/NaMt (**C**,**D**).

**Figure 6:**
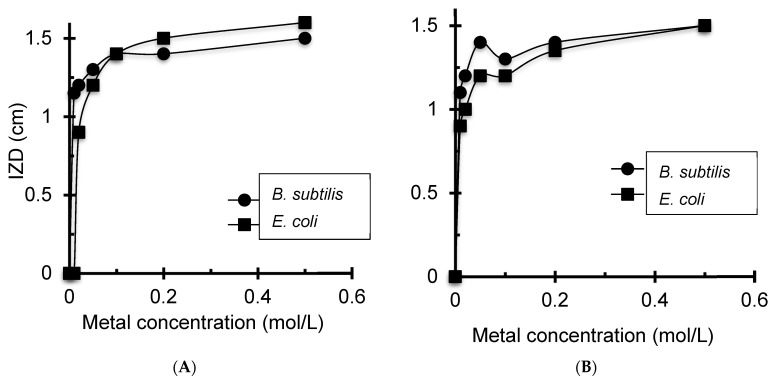
Inhibition zone diameter at different amounts of Ag^+^-loaded CMC (**A**) and CMS (**B**).

NaMt incorporation was found to slightly affect the dispersion capacity of the host-matrix, as illustrated by the slight increase in particle size (Figure 5). A possible explanation should consist in the appearance of competitive clay:polymer interaction that reduces the number of CM groups available. More than 90% of AgNPs that were dispersed displayed an average particle size not exceeding 4 nm in CMC/NaMt (Figure 5A) and 6 nm in CMC/NaMt (Figure 5B). This confirms, once again, the key roles of the biopolymer structure and density of chelating groups in metal dispersion and stabilization.

### 3.4. Antibacterial Activity

#### 3.4.1. Effect of Biopolymer Structure and Metal Incorporation

Triplicate antibacterial tests showed no antibacterial activity for the starting CMC and CMS biopolymers. A quick qualitative overview of the data obtained for modified biopolymers (Supplementary Figures S7 and S8) revealed a significant improvement in the antibacterial activity. The inhibition zone was not circular (due to the flowability of the MNP powders) and generated a low precision in graphical measurements of the inhibition zone diameter (IZD) (Supplementary Figure S6), but the use of Image-J software allowed us to reduce the standard deviation down to 1% (Supplementary Table S4). The quantitative IZD assessment showed a significant increase, providing evidence of the beneficial effect of a mere incorporation of the metal forms in agreement with previous data [6,43,44,45,46]. The relatively higher IZD values measured on *B. subtilis S168* indicate higher antibacterial activity as compared to *E. coli DH5α* for both metals in various investigated forms (Table 3). *B. subtilis* showed an IZD relatively higher compared to *E. coli*, suggesting a higher antibacterial activity on *Bacillus,* due to the Gram-positive cell wall structure with a thick peptidoglycan layer which prevents the leak of the antibacterial agent once absorbed [47]. 

The IZD for both *E. coli DH5α* and *B. subtilis S168* strains in the presence of Ag^+^/CMC reached the highest values of 4.36 and 5.40 cm, respectively. These values dropped drown to 3.81 and 4.70 cm, respectively for Ag^0^/CMC. Similar decreases were registered from 3.06 and 5.00 cm for Ag^+^/CMS to 2.54 and 3.81 cm for Ag^0^/CMS. This turns out to be general tendency for silver-modified samples regardless of the biopolymer structure and composition, which reveals a stronger antibacterial activity of oxidized silver as compared to its zero-valent form. For copper-modified samples, this tendency was not maintained, and the antibacterial activity appears to depend on the sample type and composition. The 2.24-times higher IZD values registered for Ag^0^/CMC (3.81 cm), as compared to Cu^0^/CMC (1.7 cm), account for the higher antibacterial effectiveness of metal silver, presumably due to an easier release in the liquid media. This is in agreement with the weaker retention strength of AgNP, as predicted by XPS measurements (Table 2 and Supplementary Tables S2 and S3). From these data, it appears that the IZD values for MNP-loaded biopolymers (1–5 cm) are higher than those reported for metal-loaded NaMt and metal-loaded NaMt- cellulose (1–3 cm) [6]. This almost-double diffusion zone indicates the major impact of the association of metal subnanometric particles with the polycarboxylic biocompatible materials to improve their antibacterial efficiency.

#### 3.4.2. Effects of Cation Amount and pH

The higher antibacterial effectiveness of Ag^+^/CMC and Ag^+^/CMS imposed deeper insights into the effect of cation content on their antibacterial activity (Figure 6). The latter was found to increase, as illustrated by increasing IZD with raising cation content. This increase reached a plateau at 0.2 mol/L with Ag^+^/CMC and Ag^+^/CMS for maximal antibacterial activity in both strains. This surprising result clearly shows that low Ag^+^ content is sufficiently effective to suppress the detrimental effect of the low retention capacity of CMS. This is not necessarily valid for other metals and other metal valence. 

**Figure 7:**
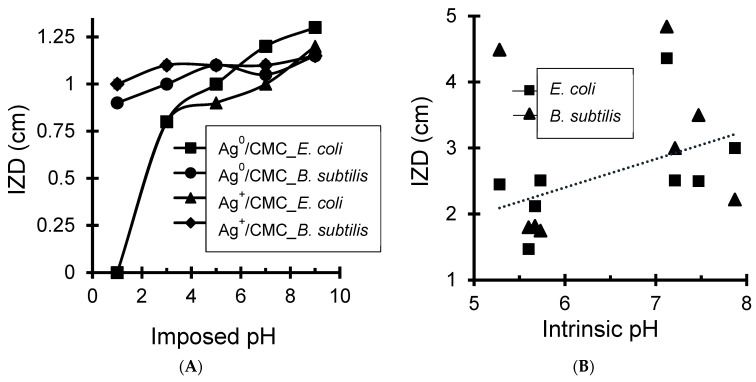
IZD dependence on imposed initial pH (**A**) and intrinsic pH of metal-loaded biopolymers (**B**).

Deeper insights into the role of pH showed a beneficial effect of increasing initial pH on the antibacterial activity, as reflected by increasing IZD for silver-modified biopolymers (Figure 7A). This result was somehow expected, given that increasing pH was already found to raise the Zeta potential, thereby reducing the material particle size. This unequivocally demonstrates the beneficial effects of the high dispersion of the antibacterial agent. The results show that the antibacterial activity is a surface phenomenon involving exchange processes between a solid surface, aqueous media and bacteria membrane.

This statement was confirmed by correlating the intrinsic initial pH induced by each antibacterial sample with the IZD (Figure 7B). Indeed, beside the few exceptions arising from the roles of the structural and chemical composition and the investigated samples, it clearly appears that samples inducing higher pH in the culture broth exhibit the highest antibacterial activity. This can explain at least partly the effect of pH, whose increase is expected to promote higher ionization of the carboxylic groups.

#### 3.4.3. Effects of Material and Metal Dispersions

**Figure 8:**
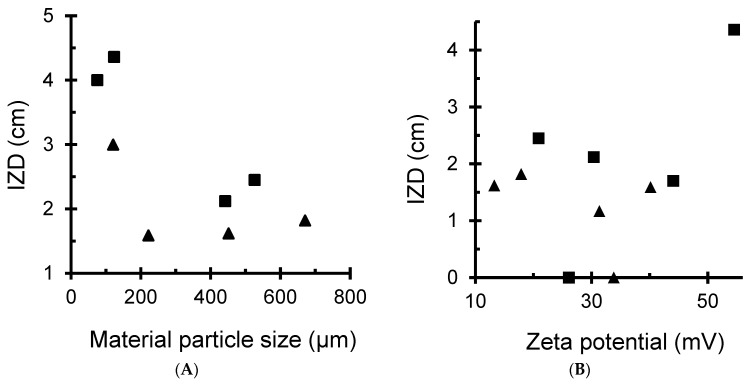
Effect of material particle size (**A**) and zeta potential (**B**) on the inhibition zone diameter (IZD). In black square: CMC-based samples, including Cu^2+/^CMC, Cu^0^/CMC, Ag^+^/CMC and Ag^0^/CMC. In black triangle: CMS-based samples, including Cu^2+/^CMS, Cu^0^/CMS, Ag^+^/CMS and Ag^0^/CMS.

The antibacterial activity was found to strongly depend on the sample particle size, polymer degree of substitution (DS) and surface charges. As expected, the highest IZD values were obtained for CMC samples with finest metal and material particle sizes (Figure 8A). The samples with increased particle size resulted in lower IZD values. The most plausible explanation resides in the higher capacity of CMC to disperse MNPs even at a subnanometric scale, as previously shown by TEM/ED-XRF (Figure 3 and Figure 4). This agrees with the general tendency on increasing IZD with increasing surface charge (Figure 8B).

**Figure 9:**
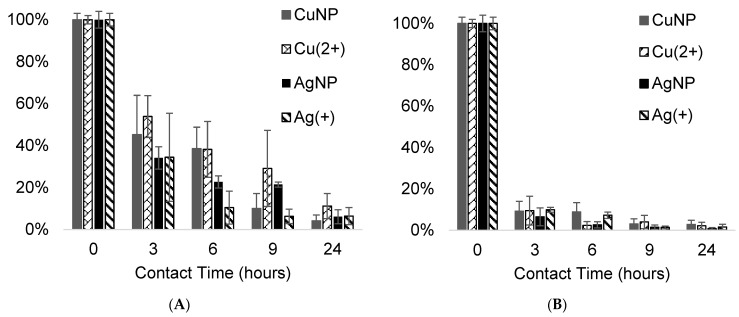
Time-course of *E. coli* viability in the presence of 0.1 mg/mL (**A**) and 1 mg/mL (**B**) of CMC samples.

The additional viability tests performed on *E. coli* with 0.1 and 1 mg/mL sample concentration showed a marked evolution in time of the antibacterial activity (Figure 9). It appears that raising the material concentration in the culture broth from 0.1 mg/mL up to 1 mg/mL induced an almost total bacteria depletion after 24 h of incubation.

NaMt-biopolymer combination exhibited lower antibacterial activity than biopolymer-based samples, as reflected by lower IZD values. This was previously explained in terms of competitive interactions between the clay mineral and polymer that involve partial involvement of CM groups in the formation of H-bridges. This should result in favorable structure expansion that allows for easy exchange with the impregnating media. However, this effect must be mitigated by the detrimental formation of bulkier MNPs, as already shown by TEM (Figure 5). An almost similar phenomenon was noticed for CMS-based samples, but with few exceptions. These exceptions may be due to the low number of CM groups, thereby demonstrating the narrow interdependence between the biopolymer DS value and amounts of both incorporated metal and clay mineral. This remains to be elucidated through deeper investigations in this direction. 

## 4. Conclusions 

The antibacterial activity induced by the incorporation of copper and silver into carboxymethyl cellulose or carboxymethyl cellulose starch appears to vary according to the metal, its valence form and biopolymer structure. The degree of substitution determines the number of carboxymethyl groups, which, in turn, governs the amount of incorporated metal, surface charges and dispersion of the metal loaded biopolymer in aqueous media according to the pH. Carboxymethyl groups act as chelating sites with specific interaction strength of their oxygen atoms according to the cationic or zero-valent form of the incorporated metal. The antibacterial activity turned out be strongly dependent on the dispersion of the modified biopolymers because of exchange processes with the surrounding bacteria. Clay mineral incorporation was found to induce detrimental competitive clay: polymer interactions that affect the dispersion of the modified biopolymers. These findings offer valuable fundamentals for designing efficient metal-loaded biopolymers for specific biomedical and biotechnological purposes.

## 5. Highlights

1. Carboxymethyl-functionalized biopolymers act as host-matrices for metal particles.
2. Carboxymethyl group density influences the metal dispersion and particle size.
3. Metal dispersion and stabilization involve interactions with matrices oxygen atoms.
4. Metal type, dispersion, valence and amount are key factors in the antibacterial activity.
5. Highly dispersed copper and silver subnanoparticles show enhanced antibacterial activity.

## Figures and Tables

**Table 1:** Some physical–chemical features of the investigated materials.

Biopolymer	Sample	Zeta Potential *		Particle Size *		Degree of Substitution **	pH *	% Error
		mV	% Error	nm	% Error			
Carboxymethyl cellulose	CMC	−33.83	5.80	289.30	1.40	0.92 + 0.01	6.37	2.10
	Cu^2+^/CMC	−13.82	1.04	526.20	3.82		5.60	0.63
	Ag^+^/CMC	−30.39	3.51	441.70	3.30		5.28	3.21
	Cu^0^/CMC	−44.05	4.40	123.10	2.40		6.37	4.32
	Ag^0^/CMC	−54.53	4.50	75.42	5.03		6.00	3.10
	CMC/NaMt	−48.36	3.80	266.60	6.04	-	6.32	2.22
	Cu^2+^/CMC-NaMt	−23.74	1.62	605.50	0.47		5.66	2.32
	Ag^+^/CMC-NaMt	−39.21	3.62	450.20	2.30		5.90	3.21
	Cu^0^/CMC-NaMt	−48.36	2.36	206.25	1.45		6.90	1.26
	Ag^0^/CMC-NaMt	−47.35	3.19	128.10	5.51		6.00	1.22
Carboxymethyl Starch	CMS	−26.11	3.50	350.20	1.50	0.51 + 0.01	6.56	3.40
	Cu^2+^/CMS	−13.28	2.21	670.60	2.45		5.67	4.32
	Ag^+^/CMS	−17.90	1.02	451.40	2.19		5.73	4.02
	Cu^0^/CMS	−31.36	1.20	221.73	1.64		6.47	2.04
	Ag^0^/CMS	−40.16	1.58	120.65	0.28		6.21	0.89
	CMS/NaMt	−33.42	6.80	299.00	2.32	-	6.41	2.11
	Cu^2+^/CMS-NaMt	−17.89	1.54	596.30	2.45		5.90	6.32
	Ag^+^/CMS-NaMt	−32.34	1.13	537.10	1.93		5.83	3.21
	Cu^0^/CMS-NaMt	−34.12	2.25	213.10	6.76		6.37	4.23
	Ag^0^/CMS-NaMt	−33.54	2.10	165.15	1.81		6.00	0.41

* Triplicate measurements were performed for of each of these features. ** The degree of substitution (DS) was defined as the number of carboxyl groups per glucose unit.

**Table 2:** Binding energy (eV) for key elements in the synthesized materials.

XPS Signal	Binding Energy (eV) *										
	CMC-Based Samples						CMS-Based Samples				
	Matrix	Alone	+Cu^2+^	+Cu^0^	+Ag^+^	+Ag^0^	Alone	+ Cu^2+^	+Cu^0^	+Ag^+^	+Ag^0^
O_1S_	O=C	530.68	530.28	529.89	530.18	529.38	530.78	530.08	529.88	530.28	529.98
	H-O-C	532.58	531.28	530.89	532.28	531.08	532.58	531.58	531.18	532.28	532.11
C_1S_	C-O-C	286.18	286.28	287.08	286.38	286.22	286.18	286.28	285.98	286.29	286.37
	C-C	284.58	284.78	284.90	284.68	284.78	284.58	284.78	284.48	284.78	284.68
	O-C=O	287.58	287.88	287.98	287.68	287.78	287.68	288.18	288.10	287.98	287.78

* The binding energy was assessed with absolute error below 0.05 eV. XPS data for pure zero-valent copper: Cu2p_3/2_ = 933 eV and for Cu^2+^ cation: Cu2p_3/2_ = 934.4 eV; XPS for pure zero-valent silver: Ag_3d5/2_ = 368.2 eV.

**Table 3:** Inhibition zone diameter (IZD) for CMC- and CMS-based samples.

Biopolymer	Incorporated Species	Samples	Inhibition Zone Diameter (IZD) (cm) *			
			*E. coli DH5α*	% Error	*B. subtilis S168*	% Error
CMC	None	CMC	0.00	0.00	0.00	0.00
	Metal cation	Cu^2+^/CMC	2.12	2.10	3.81	3.23
		Ag^+^/CMC	4.36	3.43	5.40	3.67
	Zero-valent metal	Cu^0^/CMC	1.70	5.33	4.06	1.83
		Ag^0^/CMC	3.81	1.11	4.70	4.34
	Montmorillonite	CMC/NaMt	0.00	2.32	0.00	4.23
	Metal cation	Cu^2+^/CMC/NaMt	1.50	4.32	3.90	2.36
		Ag^+^/CMC/NaMt	2.29	6.12	1.70	3.21
	Zero-valent metal	Cu^0^/CMC/NaMt	1.27	3.12	3.81	5.23
		Ag^0^/CMC/NaMt	2.11	2.10	1.27	4.21
CMS	None	CMS	0.00	2.98	0.00	3.12
	Metal cation	Cu^2+^/CMS	1.82	3.29	3.80	3.54
		Ag^+^/CMS	3.06	4.55	5.00	6.14
	Zero-valent metal	Cu^0^/CMS	3.12	6.01	4.06	4.53
		Ag^0^/CMS	2.54	1.21	3.81	2.12
	Montmorillonite	CMS/NaMt	0.00	2.04	0.00	3.24
	Metal cation	Cu^2+^/CMS/NaMt	1.00	2.32	4.50	6.55
		Ag^+^/CMS/NaMt	2.17	2.43	1.70	3.45
	Zero-valent metal	Cu^0^/CMS/NaMt	1.52	5.32	3.30	2.17
		Ag^0^/CMS/NaMt	1.20	1.00	0.20	2.91

* The inhibition zone diameter was calculated as the diameter of a hypothetical circular surface equal to the equivalent surface assessed for each sample, using Image-J software.

## Data Availability

Not applicable.

## Supplementary Materials

The following supporting information can be downloaded at: https://www.mdpi.com/article/10.3390/antibiotics11040439/s1, Figure S1. Chemical structure of sodium carboxymethyl cellulose (CMC) (a) and sodium carboxymethyl starch (CMS) (b); Figure S2. O_1s_ XPS spectra of CMC-based samples. The XPS spectra of the starting biopolymers are illustrated by red profiles. * The binding energy was assessed with absolution error below 0.05 eV; Figure S3. (A) C_1s_ XPS spectra of CMC-based samples; (B) O_1s_ XPS spectra of CMS-based samples; (C) C_1s_ XPS spectra of CMS-based samples. The XPS spectra of the starting biopolymers are illustrated by red profiles. * The binding energy was assessed with absolution error below 0.05 eV; Figure S4. Measuring the IZD graphically (a) or by using image j software (b); Figure S5. Inhibition zones in the proliferation of both bacteria strains in the presence of CMC and CMS and metal cation-loaded counterparts. The irregular IZD shape is due to the heterogenous flowability of the powders; Figure S6. Inhibition zones in the proliferation of both bacteria strains in the presence of CMC and CMS and metal zero-loaded counterparts. The irregular IZD shape is due to the heterogenous flowability of the powders; Table S1. Polarizing power (PP), ionic radius and Zeta potential of different metal cation loaded biopolymers; Table S2. XPS BE shift in CMC-based samples; Table S3. XPS BE shift in CMS-based samples; Table S4. Relative standard deviation on IZD measurements by two different methods.
