# Supplementary material for: Synthesis of Metal-Loaded Carboxylated Biopolymers with Antibacterial Activity through Metal Subnanoparticle Incorporation
Source: Antibiotics (Basel). 2022 Mar 24;11(4):439. doi: 10.3390/antibiotics11040439 (PMC9031093; doi:10.3390/antibiotics11040439)
Supplement: Supplementary file 1 [file antibiotics-11-00439-s001.zip › antibiotics-1629887-supplementary.pdf]

antibiotics

MDPI

Synthesis of metal-loaded carboxylated biopolymers with anti-bacterial activity through metal subnanoparticle incorporation

Farzaneh Noori et al…Abdelkrim Azzouz

SUPPORTING INFORMATION

Biopolymer structure

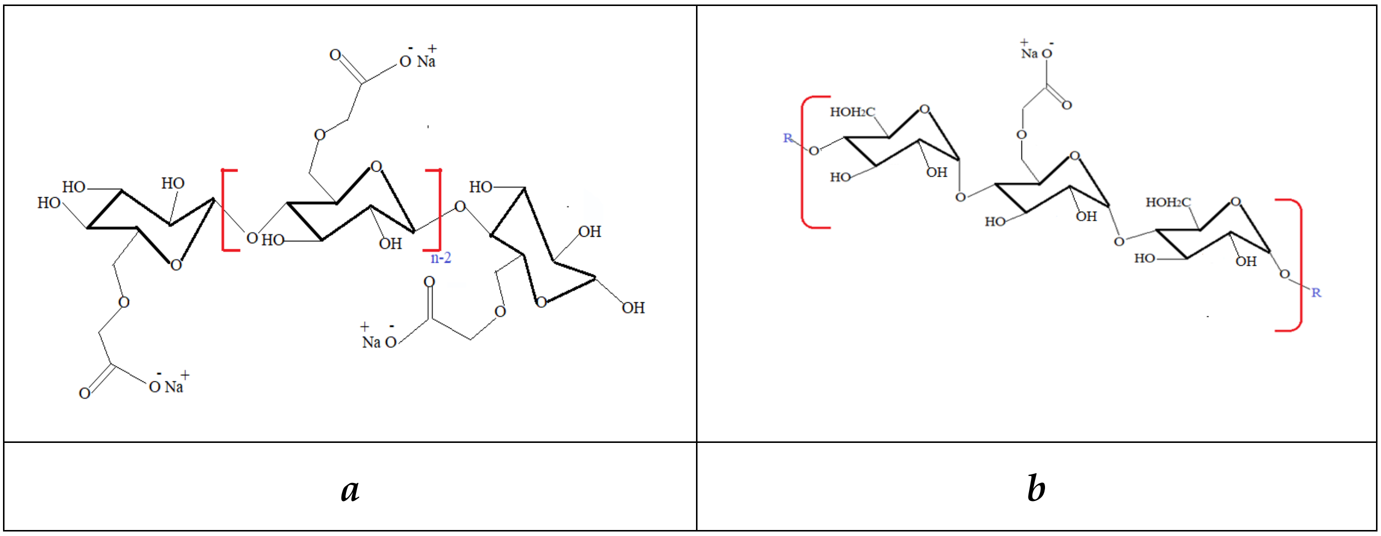

a	*b*

Figure S1. Chemical structure of sodium carboxymethyl cellulose (CMC) (a) and sodium carboxymethyl starch (CMS) (b).

Physico-chemical features of some metal cations

Table S1. Polarizing power (PP), ionic radius and Zeta potential of different metal cation loaded biopolymers

Cations	Polarizing power*	Ionic radius/pm	ZP (mV)	
			CMC	CMS
Ni^{2+}	2.80	70.	-10.05	-11.34
Cu^{2+}	2.73	73	-13.82	-13.28
Zn^{2+}	2.70	74.5	-20.90	-13.28
Co^{2+}	2.68	74	-23.10	-13.42
Pt^{2+}	2.50	80	-23.86	-15.98
Ti^{2+}	2.30	86	-24.40	-16.42
Ag^{+}	0.86	115	-30.39	-17.90
Au^{+}	0.72	137	-33.53	-21.81

The polarizing power is a cation capacity to polarize the counter anion, i.e. the capacity to attract the electron cloud anion. This factor increases with increasing charge and decreasing size of the cation.

CMC alone

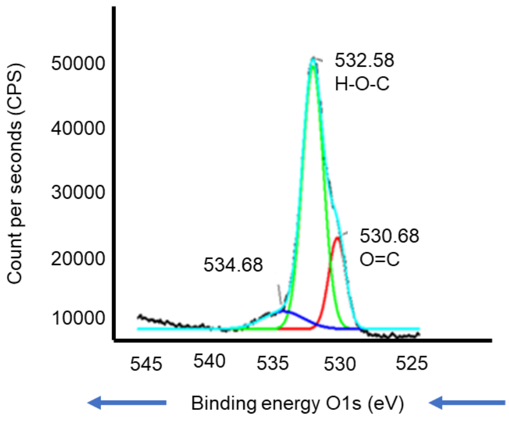

Cation-loaded CMC

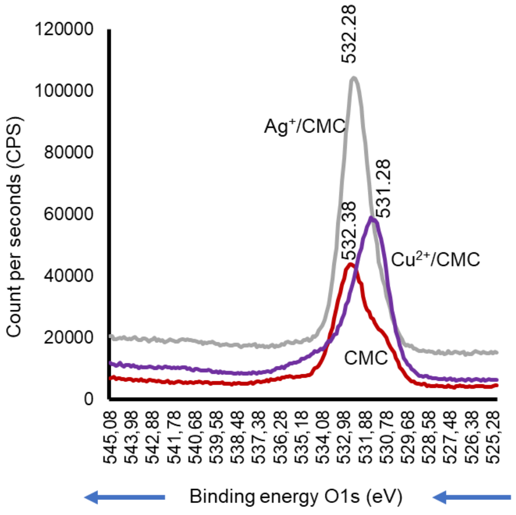

MNP-loaded CMC

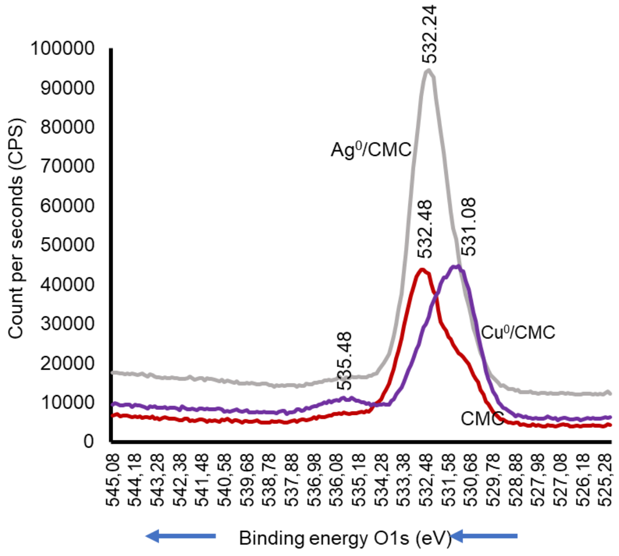

Figure S2. O1s XPS spectra of CMC-based samples. The XPS spectra of the starting biopolymers are illustrated by red profiles. * The binding energy was assessed with absolution error below 0.05 eV.

CMC alone	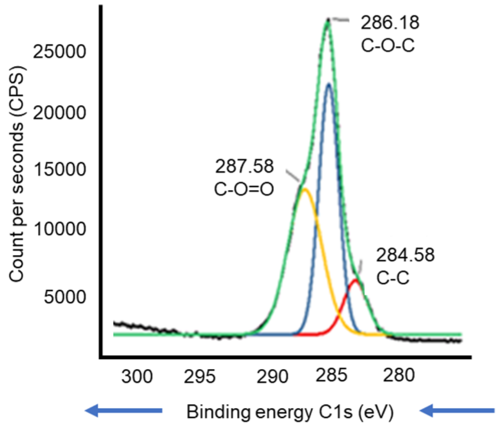
Cation-loaded CMC	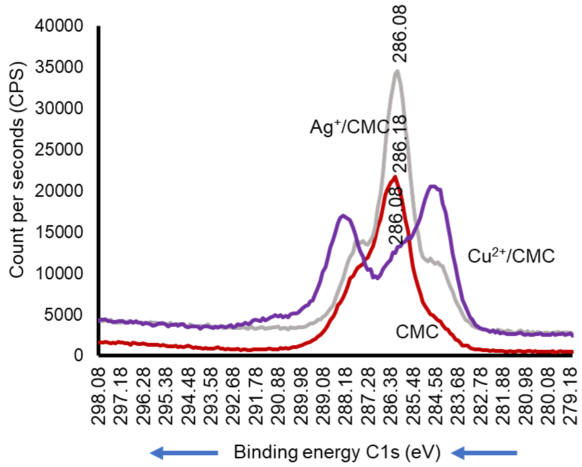
MNP-loaded CMC	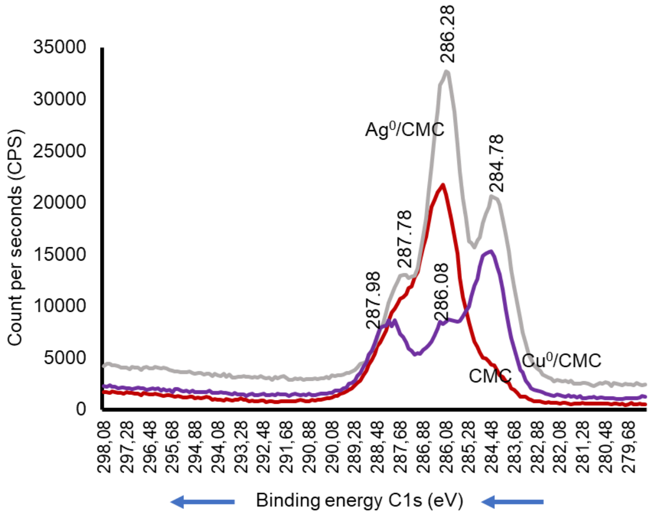

Figure S3. (A) C1s XPS spectra of CMC-based samples.

<table>
<tr><td>CMS alone</td><td>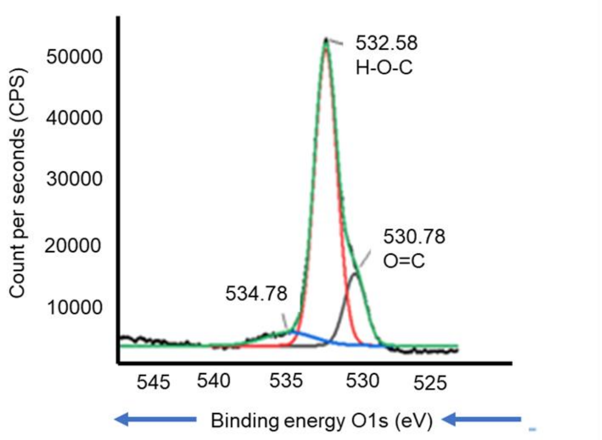
</td></tr>
<tr><td>Cation-loaded CMS</td><td>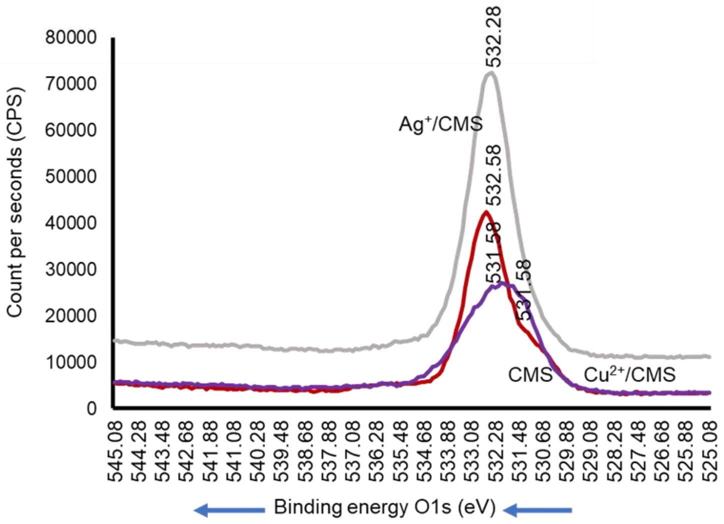
</td></tr>
<tr><td>MNP-loaded CMS</td><td>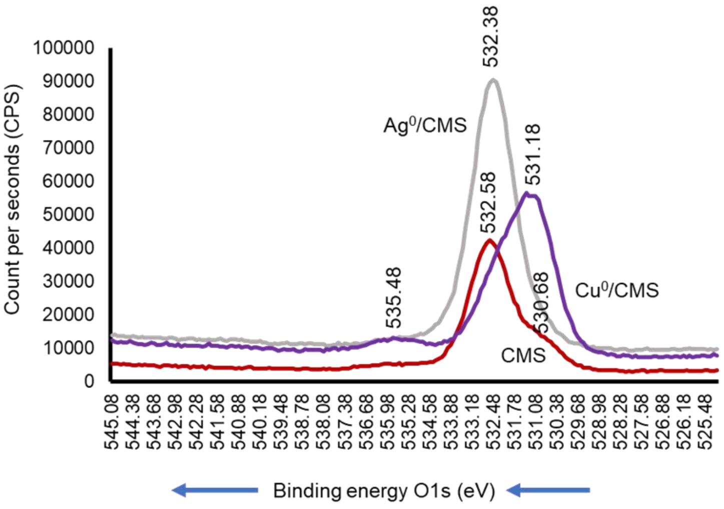
</td></tr>
</table>

Figure S3. (B) O1s XPS spectra of CMS-based samples.

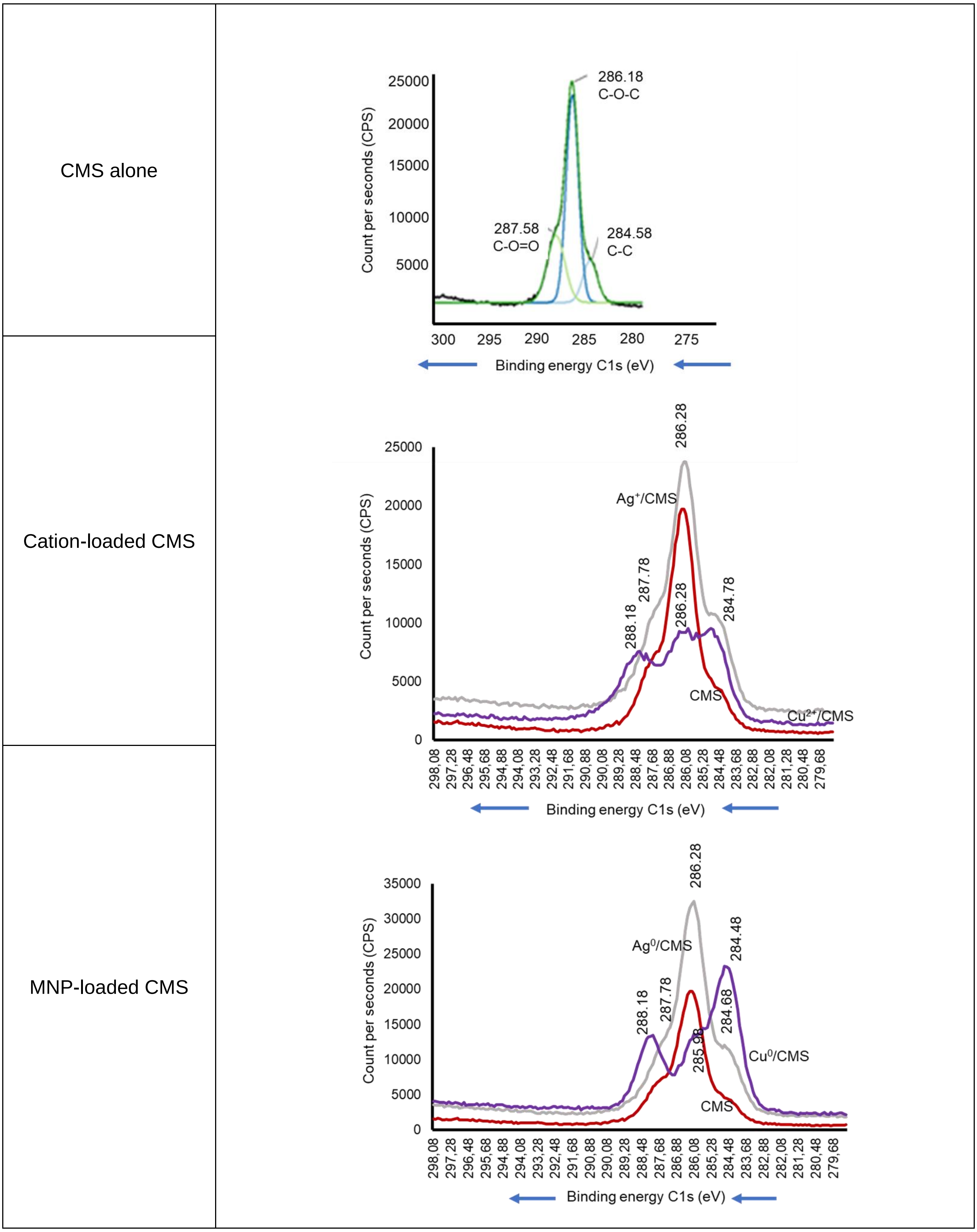

Figure S3. (C) C1s XPS spectra of CMS-based samples.

Figure S3. (A) C1s XPS spectra of CMC-based samples; **(B)** O1s XPS spectra of CMS-based samples; **(C)** C1s XPS spectra of CMS-based samples. The XPS spectra of the starting biopolymers are illustrated by red profiles. * The binding energy was assessed with absolution error below 0.05 eV.

XPS BE shift of the key atoms of the biopolymers

Table S2. XPS BE shift in CMC-based samples.

XPS signal	Binding energy (eV) of CMC- Based samples (±0.05 eV)									
	Matrix	Alone	+ Cu^{2+}	Shift* (eV)	+Cu^{0}	Shift* (eV)	+Ag^{+}	Shift* (eV)	+Ag^{0}	Shift* (eV)
O_{1S}	O=C	530,68	530.28	0.40	529.89	0.79	530.18	0.50	529.38	1.30
	H-O-C	532.58	531.28	1.30	530.89	1.69	532.28	0.30	531.08	1.50
C_{1S}	C-O-C	286.18	286.28	-0.10	287.08	-0.90	286.38	-0.20	286.22	-0.04
	C-C	284.58	284.78	-0.20	284.9	-0.32	284.68	-0.10	284.78	-0.20
	O-C=O	287.58	287.88	-0.30	287.98	-0.40	287.68	-0.10	287.78	-0.20

The BE shift was assessed as the difference between the BE values before and after metal loading.

Table S3. XPS BE shift in CMS-based samples.

XPS signal	Binding energy (eV) of CMC- Based samples (±0.05 eV)									
	Matrix	Alone	+ Cu^{2+}	Shift* (eV)	+Cu^{0}	Shift* (eV)	+Ag^{+}	Shift* (eV)	+Ag^{0}	Shift* (eV)
O_{1S}	O=C	530.78	530.08	0.70	529.88	0.90	530.28	0.50	529.98	0.80
	H-O-C	532.58	531.58	1.00	531.18	1.40	532.28	0.30	532.11	0.47
C_{1S}	C-O-C	286.18	286.28	-0.10	285.98	0.20	286.29	-0.11	286.37	-0.19
	C-C	284.58	284.78	-0.20	284.48	0.10	284.78	-0.20	284.68	-0.10
	O-C=O	287.68	288.18	-0.50	288.1	-0.42	287.98	-0.30	287.78	-0.10

The BE shift was assessed as the difference between the BE values before and after metal loading.

Procedures for IZD assessment

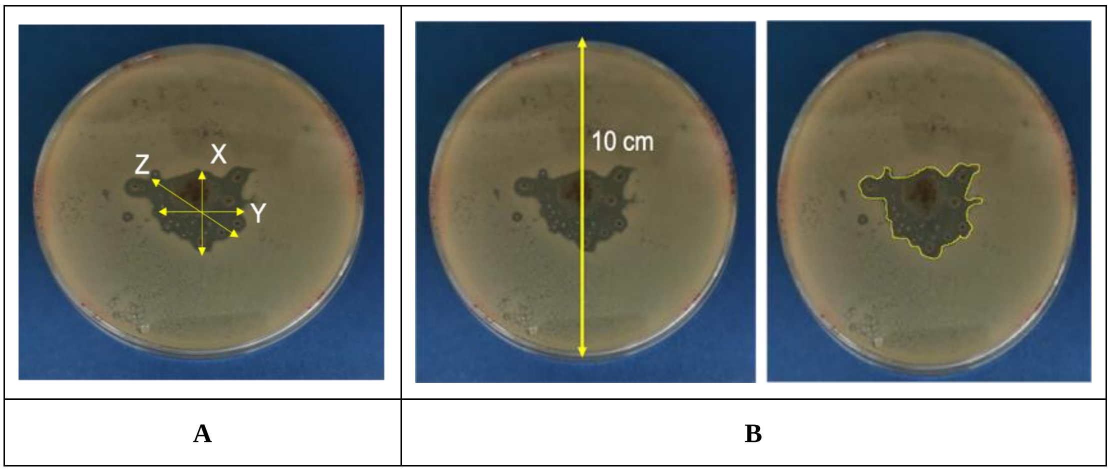

A **B**

Figure S4. Measuring the IZD graphically (a) or by using image j software (b).

Table S4. Relative standard deviation on IZD measurements by two different methods.

Samples	Standard deviation (%)			
	E. coli DH5α		B. subtilis168	
	Graphically	Image-j	Graphically	Image-j
Ag/CMS	7.5	0.5	15.5	2.5
Ag^0/CMS	5.0	1.0	9.5	2.3
Ag/CMS/NaMt	6.5	0.5	4,0	1.5
Ag^0/CMS/NaMt	0.0	0.0	0.0	0.0
Cu^2/CMS	6.0	1.5	2.0	1.0
Cu^0/CMS	2.5	0.5	1.3	0.5
Cu^2/CMS/NaMt	5.0	0.5	15.5	2.5
Cu^0/CMS/NaMt	0.0	0.0	4.5	0.6

ntibacterial tests

E. coli DH5α		*B. subtilis168*	
CMC	*CMS*	*CMC*	*CMS*
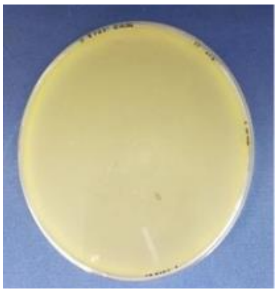	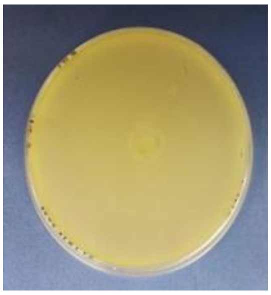	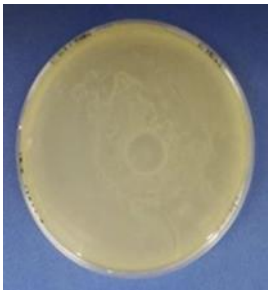	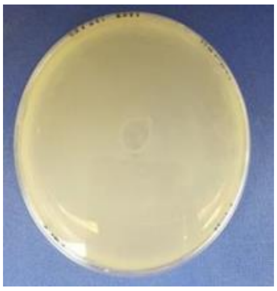
Ag^{+}/CMC	*Ag^{+}/CMS*	*Ag^{+}/CMC*	*Ag^{+}/CMS*
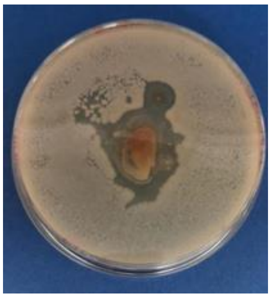	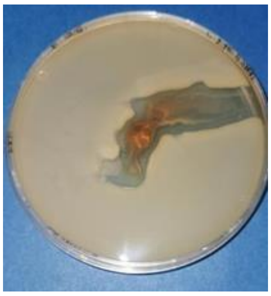	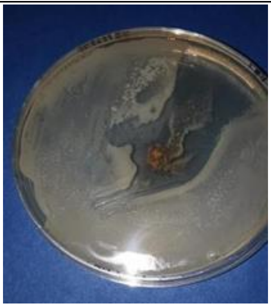	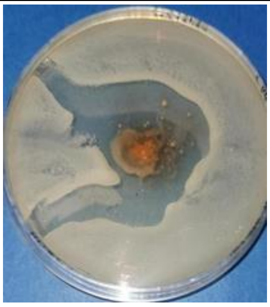
Cu^{2+}/CMC	*Cu^{2+}/CMS*	*Cu^{2+}/CMC*	*Cu^{2+}/CMS*
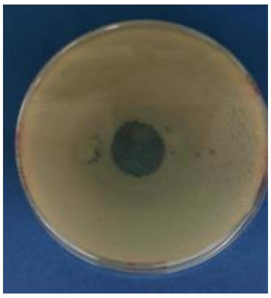	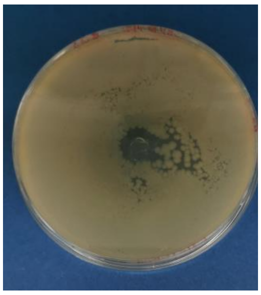	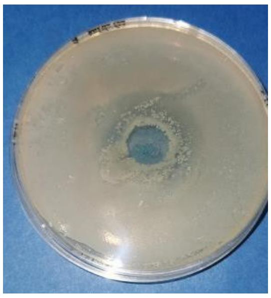	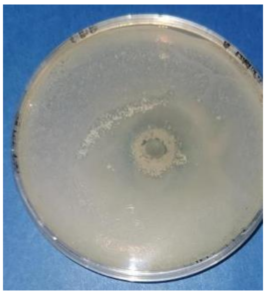

Figure S5. Inhibition zones in the proliferation of both bacteria strains in the presence of CMC and CMS and metal cation-loaded counterparts. The irregular IZD shape is due to the heterogenous flowability of the powders.

E. coli DH5α		***B. subtilis168***	
Cu^0/CMC	Ag^0/CMC	Cu^0/CMC	Ag^0/CMC
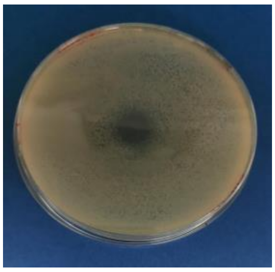	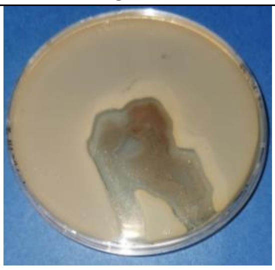	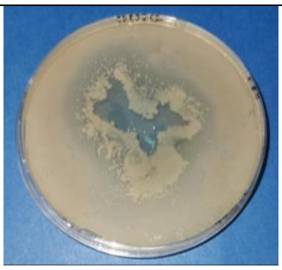	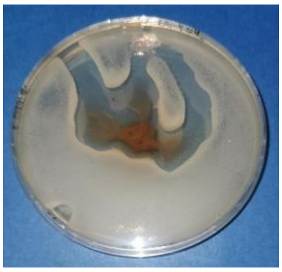
Cu^0/CMS	Ag^0/CMS	Cu^0/CMS	Ag^0/CMS
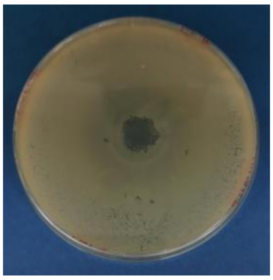	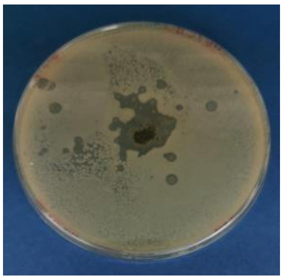	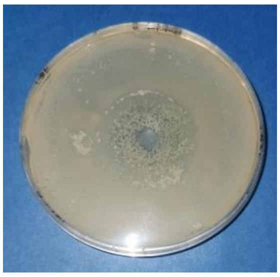	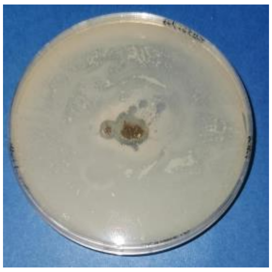

Figure S6. Inhibition zones in the proliferation of both bacteria strains in the presence of CMC and CMS and metal zero-loaded counterparts. The irregular IZD shape is due to the heterogenous flowability of the powders. .
